# Crude Glycerin Modulates the Proteomic Profile and Epithelial Adaptation of Ruminal Papillae in Lambs Fed High-Concentrate Diets

**DOI:** 10.3390/ani16091318

**Published:** 2026-04-25

**Authors:** Marco Túlio Costa Almeida, Thainara Tintori Falcão, Nicoly Leon Brun, Rafael Assis Torres de Almeida, Roberta de Lima Valença, Pedro Henrique Borba Pereira, Rodrigo de Nazaré Santos Torres

**Affiliations:** 1 Department of Animal Science, Federal University of Espírito Santo, Alegre 29500-000, ES, Brazil; roberta.valenca@ufes.br; 2 Department of Veterinary Medicine, Federal University of Espírito Santo, Alegre 29500-000, ES, Brazil; thainara.t.falcao@ufes.br (T.T.F.); nicole.l.brun@ufes.br (N.L.B.); pedro.b.pereira@ufes.br (P.H.B.P.); 3 Department of Veterinary Medicine, Federal University of Viçosa, Viçosa 36570-000, MG, Brazil; mv.rafaelassis@gmail.com; 4 Department of Animal Science, Federal University of Mato Grosso, Sinop 78060-900, MT, Brazil; santostorres_13@hotmail.com

**Keywords:** adaptive energy diet, cell mitosis, *claudin-1*, epithelial integrity

## Abstract

Crude glycerin (CG) is an energy-dense ingredient capable of partially or fully replacing corn in high-concentrate diets for ruminants. Its rapid ruminal absorption, favorable fermentative profile, and absence of lactic acid production may support safer adaptation to intensive feeding systems. The aim of this study was to evaluate the effects of replacing corn with CG (300 g/kg DM) on growth performance, feeding behavior, rumen morphometry, and proteomic responses of ruminal papillae in feedlot lambs. Sixty-five Santa Inês × Dorper lambs were assigned to either a control diet or a diet containing CG and were evaluated during pre-adaptation, adaptation, and finishing phases. Replacing corn with CG slightly reduced average daily gain (*p* = 0.02), without affecting final body weight, dry matter intake, or carcass yield (*p* > 0.05). Lambs fed CG exhibited lower subcutaneous fat thickness (*p* = 0.04) and reduced neutral detergent fiber intake during feeding behavior assessments (*p* < 0.05). Rumen papillae showed higher mitotic index and greater epithelial activity throughout the feedlot period, regardless of treatment. Proteomic analysis revealed upregulation of proteins involved in epithelial integrity (*Claudin-1*, *Occludin*) and mitochondrial energy metabolism (*ATP synthase β*, *glycerol kinase*) in CG-fed lambs, alongside downregulation of proteins related to oxidative stress and inflammation (*HSP70*, *Annexin A1*, *SOD1*, *Peroxiredoxin-6*). These findings demonstrate that CG promotes beneficial molecular adaptations in the ruminal epithelium without compromising carcass traits, supporting its use as a safe, functional, and sustainable alternative to corn in lamb finishing systems.

## Simple Summary

Crude glycerin, a coproduct of the biodiesel industry, has emerged as a safe and sustainable energy source for ruminants. In this study, lambs were fed high-concentrate diets in which corn was fully replaced by crude glycerin. Animal performance, feeding behavior, rumen health, and the molecular responses of the ruminal epithelium were evaluated. Although crude glycerin slightly reduced average daily gain, carcass yield and muscle development were not affected. In contrast, the rumen epithelium showed improved structural integrity, greater papillae density, and increased expression of proteins related to tight-junction function and energy metabolism. Proteins associated with oxidative stress and inflammation were downregulated. These findings indicate that crude glycerin promotes a favorable adaptive response in the ruminal epithelium and can be used safely and effectively as an alternative to corn in intensive lamb-finishing systems.

## 1. Introduction

High-concentrate diets are widely adopted in intensive ruminant-finishing systems because they maximize energy intake and promote faster growth [1]. Corn is widely used as an energy source in high-concentrate diets due to its high starch concentration [2]. However, starch undergoes rapid ruminal fermentation, leading to the accumulation of organic acids and a decline in ruminal pH range, which may impair rumen homeostasis [3]. When this imbalance occurs, animals become more susceptible to subacute ruminal acidosis, epithelial injury, impaired absorptive function, and metabolic stress [4]. Consequently, the search for safer energy ingredients that promote more stable rumen fermentation and support epithelial adaptation under high-concentrate conditions has intensified in recent years.

Among the alternative energy ingredients evaluated for use in high-concentrate diets, crude glycerin (CG) has gained increasing attention in ruminant nutrition. As the major coproduct of the biodiesel industry, CG is predominantly composed of glycerol, with small amounts of water, minerals, and residual methanol depending on the refining process [5]. Its use in ruminant diets is regulated to ensure minimum purity standards and safe levels of contaminants [6]. Glycerol is rapidly absorbed across the ruminal epithelium or fermented without the production of lactic acid, favoring propionate and butyrate synthesis, which reduces the risk of ruminal acidification with the low pH range commonly associated with starch feeds [7,8]. Because of these characteristics, CG has been proposed as an alternative energy source to partially replace corn in ruminant feeding systems [5,9].

Several studies have demonstrated that CG can be included in ruminant diets without impairing intake, growth performance, or carcass traits when incorporated at moderate levels [10]. In lambs, glycerin has been shown to partially replace corn while maintaining or improving energy use efficiency, depending on purity, inclusion rate, and diet composition [11]. Similar findings have been reported in feedlot cattle, where CG improves feed efficiency and alters the fatty acid profile of meat in a favorable manner [12]. Glycerin-based diets have also been associated with greater ruminal stability, reduced lactic acid accumulation, and altered fermentation patterns [8]. In addition to these productive and fermentative responses, more recent evidence highlights that glycerin-based diets may modulate metabolic responses related to mitochondrial energy metabolism and epithelial cellular activity in lambs fed concentrate-containing diets [13]. Despite these advances, most available studies have focused primarily on productive or metabolic responses, with limited attention to molecular or epithelial-level adaptations [8,12].

The ruminal epithelium plays a central role in the absorption of volatile fatty acids, maintenance of acid–base homeostasis, and preservation of barrier integrity. In addition to its absorptive function, the epithelium regulates luminal pH range through coordinated transport mechanisms and acts as a protective barrier against toxins and pathogens. Under high-concentrate feeding conditions, the increased production of fermentation end-products can challenge epithelial function, potentially leading to acidosis, epithelial damage, and impaired barrier function [14].

However, most studies evaluating glycerin supplementation have relied on productive, metabolic, or gross morphological indicators, providing limited insight into the intracellular pathways modulated by this ingredient. Proteomic analyses offer a powerful approach to identify proteins associated with epithelial integrity, oxidative stress, energy metabolism, and structural remodeling, and have been used to describe adaptive responses of the ruminal epithelium to high-energy diets and growth challenges in ruminants [15,16]. To date, however, no study has investigated the proteomic modulation of ruminal papillae in feedlot lambs fed high-concentrate diets containing crude glycerin, which restricts our ability to fully interpret the physiological consequences of replacing starch with glycerol under intensive production systems. Mitochondrial energy metabolism plays a central role in sustaining epithelial turnover and absorption processes, particularly under high-energy feeding conditions.

We hypothesized that substituting corn with crude glycerin in high-concentrate diets would modulate key metabolic and structural pathways of the ruminal epithelium, promoting adaptive responses related to barrier function, epithelial turnover, and mitochondrial energy metabolism. The inclusion level of crude glycerin used in this study was selected based on previous research demonstrating its effectiveness and safety in ruminant diets. Therefore, this study was designed as a mechanistic approach, focusing on the proteomic and epithelial responses rather than evaluating dose–response effects. Therefore, the objective of this study was to evaluate the effects of crude glycerin inclusion (300 g/kg DM) on growth performance, feeding behavior, rumen morphometry, and the proteomic profile of ruminal papillae in feedlot lambs. By integrating productive, morphological, and molecular analyses, this study provides novel insights into the epithelial adaptations associated with replacing starch with glycerol in intensive lamb-finishing systems.

## 2. Materials and Methods

The study was conducted at the Experimental Farm of the Federal University of Espírito Santo (UFES) in Alegre, Espírito Santo, Brazil (latitude: 20°44′41.78″ S; longitude: 41°29′13.67″ W). All experimental procedures were approved by the Institutional Animal Care and Use Committee of UFES (protocol no. 005/2022).

### 2.1. Animals, Treatments, and Experimental Design

Sixty-five uncastrated crossbred lambs (Santa Inês × Dorper), approximately 90 days old, weighing 18.32 ± 2.59 kg, were randomly allocated to individual indoor pens (1.5 m^2^) and distributed in a randomized block design based on initial body weight (BW). The animals were assigned to one of two dietary treatments and subjected to a pre-adaptation, adaptation, and finishing period in a feedlot lasting approximately 60 days. The treatments consisted of: CON (control; without crude glycerin) and G300 (300 g/kg crude glycerin on a DM basis), Table 1.

Initially, the lambs underwent a 15-day pre-adaptation period during which they were fed a basal diet composed of corn silage, concentrate, and a mineral mixture offered ad libitum (Table 1). This period allowed the stabilization of voluntary feed intake and the rumen microbiota prior to the experimental treatments. At the end of the pre-adaptation phase, five lambs weighing 18.71 ± 0.65 kg were randomly slaughtered to establish a baseline for subsequent comparisons at the beginning of the experimental period (day 0).

After the pre-adaptation period, the animals were assigned to two experimental groups (CON, n = 30, 19.22 ± 1.25 kg BW; G300, n = 30, 18.99 ± 1.63 kg BW) and submitted to a 14-day adaptation period to high-concentrate diets. Three step-up diets were offered during this phase, progressively increasing the concentrate level on a dry matter (DM) basis: four days on diet 1 (520 g/kg DM concentrate), five days on diet 2 (640 g/kg DM concentrate), and five days on diet 3 (760 g/kg DM concentrate). The final finishing diet contained 880 g/kg of DM of concentrate (Table 1). At the end of the 14-day adaptation period, ten lambs from each group (CON, 21.88 ± 1.85 kg BW; G300, 21.42 ± 2.65 kg BW) were randomly selected for slaughter and detailed evaluation.

The lambs that remained in the finishing period continued to receive their respective diets (CON, n = 20; G300, n = 20) until they reached a body weight (BW) of approximately 35 kg, at which point they were slaughtered.

The experimental diets were formulated to be isonitrogenous (177 g/kg CP) and isocaloric (2.7 Mcal/kg ME), following the recommendations of NRC (2007) [17]. The diets consisted of corn silage, ground corn, soybean hulls, soybean meal, urea, a premix mineral–vitamin, limestone, and dicalcium phosphate (Table 1). The crude glycerin (CG) was sourced from a commercial soybean and sunflower processing plant and contained 82.5% glycerol and less than 0.01% methanol.

The experimental diets were offered to the lambs ad libitum twice daily (0800 h and 1600 h) and adjusted to allow approximately 10% of feed refusals. The concentrate mixtures were pre-formulated, and the roughage and crude glycerin were added and homogenized at the time of feeding to increase voluntary intake and minimize feed selection by the lambs. Water was provided ad libitum throughout the experimental period.

### 2.2. Experimental Procedures and Sample Collection

Throughout the experimental period, feed offered and refusals were weighed daily to estimate dry matter (DM) and neutral detergent fiber (NDF) intake. Lambs were weighed at the end of each experimental phase, and during the finishing period, they were weighed every 15 days to monitor growth until reaching slaughter weight. Average daily gain (ADG) was calculated using body weights obtained after a 16 h fasting period by dividing the difference between the final and initial weights by the number of days in the finishing phase.

The ingestive behavior of the lambs was evaluated at three time points during the finishing period: at the beginning (day 7), midpoint (day 21), and end (day 42) of the finishing period. During the entire ingestive behavior evaluation period, five previously trained observers monitored the animals continuously for 24 h, resulting in a total of 72 h of behavioral evaluation across the experiment. Behavioral activities were recorded at five-minute intervals and included feeding, rumination, and idling, following the methodology described by Almeida [18].

Rumen samples were collected throughout the experimental period as the animals were slaughtered. Five lambs were randomly selected and slaughtered at the end of the pre-adaptation period (day 0), 20 at the end of the adaptation period (day 14), and the remaining 40 animals when they reached a body weight (BW) of approximately 35 kg. All animals underwent feed withdrawal with free access to water for a 16 h period. The lambs were stunned using a captive bolt pistol (model CASH Special, Accles & Shelvoke Ltd., Birmingham, UK) activated by an explosive cartridge and subsequently bled by severing the carotid arteries and jugular veins, following the procedures recommended by Brazilian legislation [19].

After evisceration, the compartments of the digestive tract (rumen, reticulum, omasum, and abomasum) were emptied, washed, and weighed individually. Carcasses were weighed 45 min postmortem at room temperature to determine hot carcass weight (HCW) and then stored in a cold chamber at 4 °C for 24 h. The carcasses were suspended by the gastrocnemius tendons on hooks spaced 17 cm apart to determine cold carcass weight (CCW), which was subsequently used for quantitative carcass evaluation.

The rumen was visually examined for lesions using a scoring scale ranging from 0 to 10, in which a score of 0 indicates the absence of lesions and a score of 10 represents severe ruminal lesions, according to the method described by Bigham and McManus [20].

Ruminal tissue samples were collected from the base of the left ventral sac, rinsed with phosphate-buffered saline (PBS; 0.1 M, pH 7.4), and preserved in paraffin. For histomorphometric analysis, a 1 cm^2^ section of ruminal tissue was collected, fixed in 10% neutral buffered formalin for 48 h, and subsequently embedded in paraffin for later measurements [21]. For proteomic analyses, rumen papillae with normal and visually healthy appearance were selected, rinsed with PBS, placed into 2 mL cryotubes, and immediately stored in an ultra-freezer at −80 °C until analysis.

### 2.3. Laboratory Analyses and Calculations

Prior to chemical analysis, samples of feed and refusal were pre-dried in a forced-air oven (Tecnal^®^, model TE394-2, Piracicaba, SP, Brazil) at 55 °C until constant weight for 72 h at the Animal Nutrition Laboratory of the Federal University of Espírito Santo. Composite samples of feed and refusal were then ground through a 1 mm screen in a Wiley knife mill (Marconi^®^, model MA340, Piracicaba, SP, Brazil) for the determination of dry matter (DM; method 934.01), according to the Association of Official Analytical Chemists [22]. Neutral detergent fiber (NDF) was determined according to [23].

Hot carcass weight yield (1), cold carcass weight (2), and cooling losses (3) were determined using the following equations:

(1) $$HCY\left(\%\right)=\left(\frac{HCW}{BWS}\right)\times 100$$

(2) $$CCY\;(\%)=\left(\frac{CCW}{BWS}\right)\times 100$$

(3) $$CL\;(g/kg)=\left(\frac{HCW-CCW}{HCW}\right)$$

where: HCY: hot carcass yield; HCW: hot carcass weight; BWS: body weight; CCY: cold carcass yield; CCW: cold carcass weight; CL: cooling loss.

The measurements obtained from the *Longissimus* muscle between the 12th and 13th thoracic ribs were used to calculate the loin eye area (LEA), following the method described by [24]. The equation used was:

(4) $$LEA\;({cm}^{2})\;=\;\left(\frac{A}{2}\times \frac{B}{2}\right)\times \pi$$

where: A: maximum length (cm) of the longissimus muscle; and B: maximum depth (cm) of the *Longissimus* muscle.

The thickness of the subcutaneous fat over the 12th rib was measured 11 cm from the back–loin line using a digital caliper (Mitutoyo Sul Americana, Jundiaí, SP, Brazil).

Ruminal morphometry was performed on ten papillae per animal, comprising five papillae from visually healthy regions and five from damaged regions. The measurements obtained included the average number of papillae per cm^2^, mean papilla area, total absorptive surface area per cm^2^, and the relative participation of papillae in the total absorptive surface area, as described by [25]. The mitotic index was determined according to the procedure described by [26].

Proteomic analysis was performed to identify the proteins expressed in the ruminal epithelium of lambs at a commercial analytical laboratory. Rumen papillae were macerated in liquid nitrogen, lyophilized, and subjected to protein extraction using a cell lysis buffer (pH 8.0) containing urea (8 M), NaCl (75 mM), Tris-HCl (50 mM), and protease inhibitors. The extracted proteins were dialyzed and quantified using the EZQ^™^ Protein Quantification Kit (Molecular Probes^®^, Eugene, OR, USA). Proteins were then digested with trypsin, and the resulting peptides were purified using ZipTip^®^ C18 pipette tips (Millipore, Burlington, MA, USA).

The analyses were performed using a mass spectrometer (Shimadzu^®^ LC-IT-ToF/MS, Kyoto, Japan), coupled to a high-performance liquid chromatography system (HPLC; Shimadzu Prominence UFLC XR, Kyoto, Japan). The instrument was equipped with a Shim-pack XR-ODS C18 analytical column and operated in positive electrospray ionization (ESI) mode with a mass-to-charge (*m*/*z*) scan range of 200–4000. The MS/MS spectra obtained were converted to MGF format and processed using Mascot^®^ software version 2.6 (Matrix Science, London, UK). Database searches were performed against the SwissProt and NCBI protein databases restricted to mammalian species. Only ions with Mascot scores above the significance threshold (*p* < 0.05) were considered confidently identified.

Protein functional identification was performed using the UniProtKB/Swiss-Prot^®^ database. Protein–protein interaction networks were constructed using STRING^®^ v12.0, applying a high-confidence interaction score (>0.7). Functional enrichment was assessed based on Gene Ontology (GO) biological processes and Kyoto Encyclopedia of Genes and Genomes (KEGG) metabolic pathways.

### 2.4. Statistical Analysis

The data were analyzed by ANOVA using the MIXED procedure of Statistical Analysis System (version 9.4; SAS Institute Inc., Cary, NC, USA), with adjustments made for repeated measurements over time. The statistical model included the fixed effects of treatment (Control and G300) and time (pre-adaptation, adaptation, and finishing of the feedlot period). The interaction treatment × time was evaluated for the performance of feedlot lambs. The covariance structure was defined based on the Akaike (AIC) and Schwarz (BIC) information criteria; the structure that provided the best fit to the model was adopted. Block effects were included as random effects. Adjusted means were compared using a Tukey test at a significance level of α = 0.05, and values between 0.10 and 0.05 were interpreted as a trend toward significance.

## 3. Results

### 3.1. Performance, Ingestive Behavior, Characteristics of the Carcass, and Gastric Compartments

The replacement of corn with CG in the finishing diets of lambs did not affect fasting BW (*p* > 0.05). A treatment × time interaction was observed for ADG (*p* = 0.02), indicating that the response to crude glycerin varied across the feedlot periods. DMI and FE were not influenced by treatments (*p* > 0.05), with average values of 1.065 kg/day and 0.270 kg/kg, respectively (Table 2).

**Figure 1:**
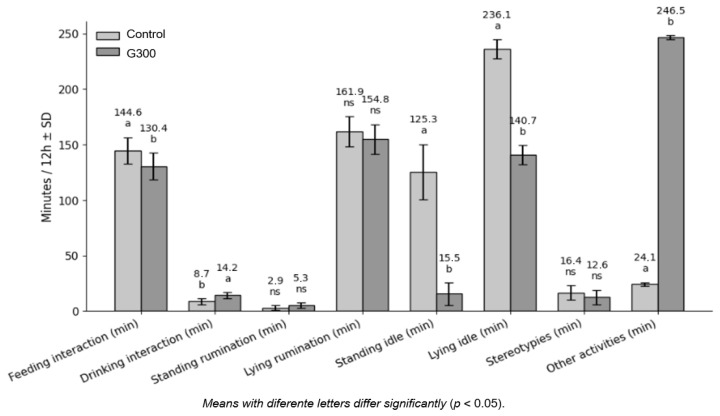
Ingestive behavior parameters of feedlot lambs fed diets containing 300 g/kg of corn (CON) or 300 g/kg of crude glycerin (G300) in the dry matter of the total mixed ration. ^a^, ^b^ Values within a row with different superscripts differ significantly at *p* < 0.05 (Tukey test). ns, not significant.

Ingestive behavior parameters (Figure 1) indicated a reduction in feeding time (*p* > 0.05) for lambs fed diets containing 300 g/kg of CG, along with an increase in drinking time (*p* < 0.05). The differences corresponded to approximately 10% less time interacting with the feed bunk and 60% more time at the water trough, respectively, compared with lambs fed only corn in the TMR.

Lambs in the CON group spent more time idling (*p* < 0.05), both standing (CON = 125.3 min; G300 = 15.55 min) and lying (CON = 236.1 min; G300 = 140.7 min). Rumination time, either standing or lying, did not differ between treatments (*p* > 0.05).

Stereotypic behaviors were not affected by treatments (*p* > 0.05), whereas the time spent on other activities was substantially greater for lambs fed 300 g/kg of CG (246.5 vs. 24.1 min, *p* > 0.05), representing more than a 900% increase compared with the control group.

**Figure 2:**
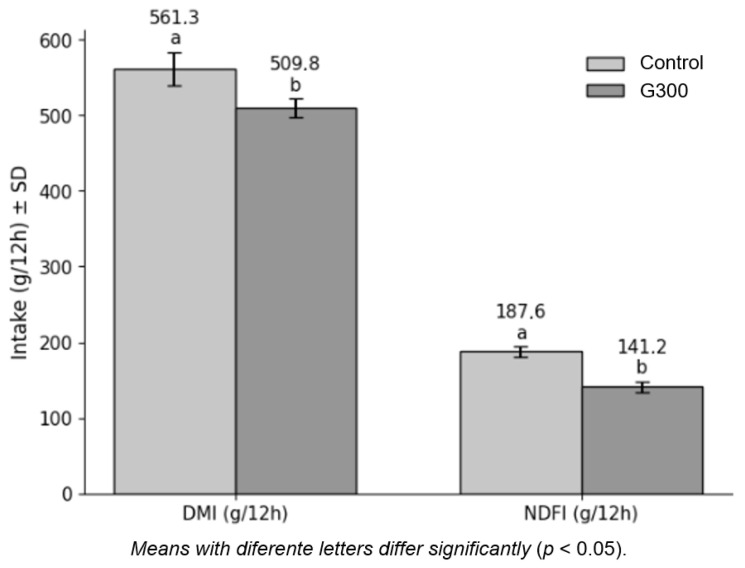
Dry matter intake (DMI) and neutral detergent fiber intake (NDFI) during the 12 h period of ingestive behavior of feedlot lambs fed diets containing 300 g/kg of corn (CON) or 300 g/kg of crude glycerin (G300) in the dry matter of the total mixed ration. ^a^, ^b^ Values within a row with different superscripts differ significantly at *p* < 0.05 (Tukey test).

The evaluation of feed intake during the 12 h period of ingestive behavior observation (Figure 2), corresponding to the interval between morning (0600 h) and afternoon (1800 h) feedings, showed that lambs fed diets containing 300 g/kg of CG had lower DMI and NDFI compared with the control group (*p* < 0.05). DMI decreased from 561.3 g/12 h to 509.8 g/12 h, whereas NDFI decreased from 187.6 g/12 h to 141.2 g/12 h.

Regarding carcass yield and traits (Table 3), the replacement of 300 g/kg of CG in the diet tended to reduce carcass chilling loss (*p* = 0.07). Subcutaneous fat thickness at the 12th rib was affected (*p* = 0.02), with higher values observed in lambs fed 300 g/kg of corn (4.6 mm) than in those receiving 300 g/kg of CG (3.7 mm) in the finishing diets. This result indicates a reduction in subcutaneous fat deposition without affecting carcass yield or conformation. No differences were observed among treatments (*p* > 0.05) for empty body weight, hot and cold carcass weights and yields, or *Longissimus* muscle area.

The inclusion of crude glycerin affected rumen weight (*p* < 0.05), whereas reticulum, omasum, and abomasum weights were not influenced by diet (*p* > 0.05). All gastric compartments increased in weight over time (Table 4; *p* < 0.001). In contrast, the ruminal lesion score showed for both treatment (*p* = 0.07) and time (*p* = 0.09), with numerically greater values observed during the finishing phase, when animals remained longer under a high concentrate diet. The incidence of liver abscesses was low across all phases; however, a treatment effect was observed (*p* = 0.04).

### 3.2. Morphometry and Proteomics

The number of ruminal papillae (NOP; *p* = 0.03) and the mitotic index (MIT; *p* = 0.01) increased throughout the feedlot period, reflecting the effect of time rather than crude glycerin inclusion, with a tendency for the total absorptive surface area (ASA, cm^2^; *p* = 0.09). These results indicate papilla development and proliferative activity increased over time during the feedlot period (Table 5). The average papilla area (APA) and papillary area proportion (PA) did not differ among treatments or periods (*p* > 0.05).

The use of 300 g/kg of CG in the diet of lambs resulted in significant changes in the ruminal epithelial proteomic profile (Table 6). Differentially expressed proteins were identified, mainly related to cell integrity, energy metabolism, and oxidative stress response. Among the proteins associated with epithelial integrity, the expression of *Claudin-1* (log_2_ FC = 1.4; *p* < 0.001) and *Occludin* (log_2_ FC = 1.0; *p* = 0.01) was increased, resulting in upregulation of genes associated with cell integrity. The *ATP synthase subunit β* showed higher relative abundance (log_2_ FC = 0.8; *p* = 0.04), indicating enhanced mitochondrial metabolism. *Glycerol kinase* exhibited the greatest upregulation (log_2_ FC = 2.3; *p* < 0.001), reflecting activation of metabolism pathways related to glycerol utilization. Conversely, proteins associated with cellular stress were downregulated in the G300 group, including *HSP70* (log_2_ FC = −1.2; *p*= 0.01), *Annexin A1* (log_2_ FC = −0.9; *p* = 0.03), and *SOD1* (log2 FC = −0.6; *p* = 0.01), whereas *Cytokeratin 4* did not differ significantly between treatments (*p* > 0.05).

**Figure 3:**
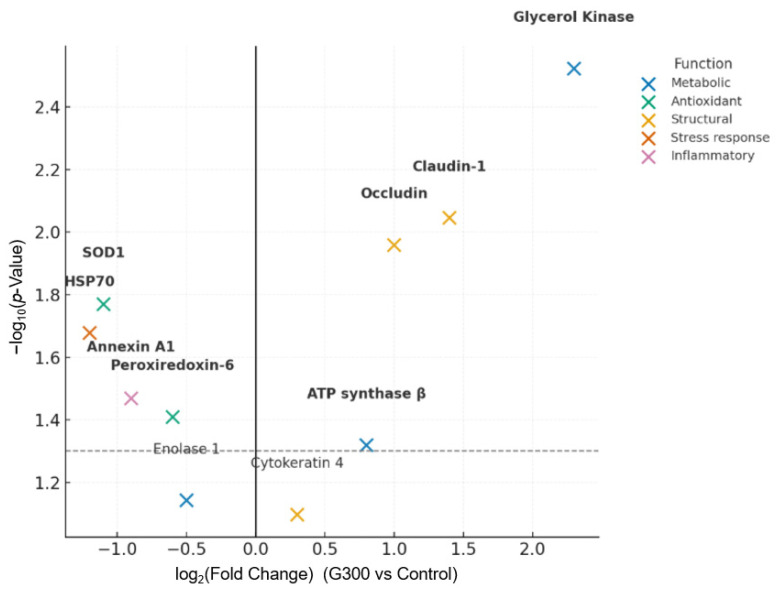
Volcano plot of differentially expressed proteins in the ruminal epithelium of lambs fed diets containing 0 (Control) or 300 g/kg of crude glycerin in dry matter.

Each dot represents a protein identified by LC-MS/MS, with the x-axis showing the log_2_ (fold change) and the y-axis showing the –log_10_ (*p*-value). Colored dots indicate proteins grouped by biological function: metabolic, antioxidant, structural, inflammatory, and stress response. Dashed horizontal and vertical lines represent significance (*p* < 0.05) and fold change thresholds, respectively. Highlighted proteins include *Glycerol Kinase*, *Claudin-1*, *Occludin*, *ATP synthase β*, *HSP70*, and *SOD1* (Figure 3). 

## 4. Discussion

In the present study, the inclusion of CG did not affect DMI, final BW of growing lambs, and consequently did not affect final BW or fasting BW. Some authors, however, have reported opposite results regarding the partial or total replacement of corn with CG in high-concentrate diets for feedlot lambs, with negative effects on DMI and animal performance [27,28,29]. Similar responses have also been observed in feedlot beef cattle fed CG derived from corn [30,31] and soybean oil [32].

The energy concentration, i.e., gross energy (GE), of CG may vary by up to 20% compared with corn, with reported values of 14.5 MJ/kg DM for GE of CG [10] and approximately 18.2 MJ/kg DM for GE of corn [17,33]. Despite differences in energy composition compared with corn, crude glycerin can provide fermentable substrate and metabolizable energy for growing lambs. Thus, in finishing diets for growing lambs, the complete replacement of ground corn with CG may contribute to maintaining fermentable energy supply for microbial activity; however, this inference is based on performance and literature, as ruminal fermentation parameters were not directly measured in the present study. However, the reduction in average daily gain observed in the present study suggests that the complete replacement of corn with crude glycerin may not fully sustain growth performance under these conditions. This discrepancy may be related to differences in energy utilization, metabolic partitioning, or feeding behavior.

Regarding ruminal fermentation, previous studies have reported that CG does not negatively affect short-chain fatty acid (SCFA) production; however, these parameters were not measured in the present study. CG does not negatively affect short-chain fatty acid (SCFA) production, ruminal ammonia nitrogen concentration, or ruminal pH range. However, the acetate/propionate ratio may be altered depending on the level of CG inclusion [29]. Propionate is extensively metabolized in the liver during the postprandial period, contributing to ATP synthesis via gluconeogenesis and participating in satiety signaling pathways, which may regulate DMI [34]. The ruminal metabolism of CG favors propionate production without causing excessive acidification of the ruminal environment, thereby enhancing glucose supply to the animal while maintaining stable pH conditions.

In high-concentrate lamb diets, starch fermentation typically results in increased propionate production, as well as lactate accumulation [35]. Excessive lactate production reduces ruminal pH range [36], compromising ruminal epithelial structure and absorptive function. Under such conditions, ruminal inflammation (e.g., rumenitis) can reduce feed intake, performance, and feeding behavior due to digestive discomfort. Therefore, the use of CG in feedlot diets may help prevent reductions in ruminal pH and contribute to a more stable fermentative environment and improved ruminal comfort. Although lesion scores remained within ranges commonly reported for high-concentrate diets, the tendency for increased ruminal lesion scores, together with the occurrence of hepatic abscesses, suggests that the high fermentative challenge of the finishing diet may have contributed to epithelial stress. This response may partially explain the reduction in average daily gain observed in lambs fed crude glycerin.

The behavioral patterns observed herein may be partly explained by reduced idleness in lambs fed CG. Animals in the control diet may have experienced digestive discomfort caused by physical distension and gas accumulation from starch fermentation, which can lead to restlessness and attempts to adjust to the altered ruminal environment. Similar behavioral responses have been reported in animals exposed to diets with lower palatability or increased metabolic challenge [31,37]. It is important to highlight that, although soybean hulls contribute to fiber supply, their physical effectiveness is lower compared to long-particle forages. Thus, future studies should consider the inclusion of physically effective fiber sources to optimize rumination and salivation under high-concentrate feeding conditions.

In the present study, animals fed CG exhibited reduced idleness and altered ingestive behavior. These results are consistent with previous findings reported by Eiras et al. [38], suggesting that glycerin inclusion may influence energy availability and behavioral patterns. This effect may reflect differences in energy utilization, derived from both ruminal fermentation and hepatic metabolism of glycerol, ultimately resulting in greater overall energy availability and more time allocated to other activities during the evaluation period.

Overall, the results indicate that the diet containing 300 g/kg of crude glycerin altered ingestive behavior and reduced idleness, modifying the natural behavioral pattern of feedlot lambs. These findings reinforce the importance of considering not only productive performance but also behavioral and physiological adaptation aspects when incorporating alternative ingredients into finishing diets.

The influence of propionate metabolism on lipid deposition should be considered, as propionate is a more efficient substrate for adipogenesis compared with acetate. The increased hepatic gluconeogenic flux arising from CG fermentation may enhance fat deposition efficiency, contributing to improved carcass characteristics in lambs fed glycerin-based diets.

Regarding carcass characteristics, the replacement of corn with CG in the diet did not affect the main quantitative carcass traits, such as carcass weight and hot and cold carcass yield. However, the induction of glycerin in the lambs diet significantly changes the qualitative fat indicators. This reduction suggests that glycerin may alter the dynamics of subcutaneous fat deposition.

Considering that glycerin is rapidly fermented to propionate in the rumen, increasing the gluconeogenic flux and reducing the acetate/propionate ratio, it is plausible that this metabolic shift favors lower de novo fatty acid synthesis in adipose tissue [39,40]. Since fatty acid synthesis in ruminant adipose tissue depends primarily on acetate, and to a lesser extent on butyrate [41], the change in the ruminal fermentation profile caused by glycerin inclusion may reduce subcutaneous lipogenesis. In this context, increased propionate production may shift energy metabolism toward gluconeogenesis rather than lipid synthesis. Consequently, the lower availability of lipogenic precursors may result in reduced external fat deposition, without adverse effects on muscle mass, as evidenced by the similarity in *Longissimus* muscle area among treatments.

Animals receiving the conventional diet exhibited greater feed intake during the behavioral observation period (Figure 2), although total dry matter intake did not differ between treatments (*p* > 0.05). This difference in short-term intake may contribute to greater rumen distension and stimulation of rumen wall development. Additionally, higher intake of fermentable substrate intensifies microbial activity and fermentation rates, favoring growth of the compartment. Thus, the higher relative weight of the rumen in the control group reflects the higher consumption observed during the adaptation period, which reinforces the influence of intake level on forestomach development. This effect was restricted to the rumen, as no differences were observed for the reticulum, omasum, and abomasum, indicating a compartment-specific response to crude glycerin inclusion.

The protein profile of ruminal papillae provides insights into the effects of crude glycerin supplementation on ruminal health. In the pharmaceutical industry, glycerin is commonly used for skin hydration and for the repair of microlesions in epidermal tissue due to its barrier-protective properties [42]. However, in animal science, there is currently limited information regarding the effects of crude glycerin on the cellular activity of ruminal papillae in lambs. In the present study, proteomic analysis revealed modulation of proteins associated with epithelial integrity, energy metabolism, and reduced cellular stress.

Among the proteins evaluated in the present study, *ATP synthase β* is an enzyme associated with cellular metabolism and is responsible for ATP synthesis [43]. Under nutritional conditions in which diets for growing lambs contain high concentrations of rapidly digestible carbohydrates, such as corn and CG, the ruminal papillae epithelium requires a high energy supply to sustain processes, including volatile fatty acid absorption and epithelial cell turnover. Thus, lambs fed CG exhibited increased expression of proteins associated with energy metabolism in ruminal papillae cells, indicating enhanced cellular metabolic activity. During the feedlot period, ruminal papillae development changed over time, as evidenced by the increase in papilla number and mitotic index, regardless of crude glycerin inclusion. In contrast, proteomic responses may suggest improved epithelial functional adaptation, although papilla morphometric parameters were not affected by treatment.

This effect may be complemented by the expression and activity of the proteins *Claudin-1* and *Occludin*, which are classified as tight junction proteins and are responsible for maintaining epithelial barrier integrity, regulating membrane permeability, and preserving ruminal epithelial structure [44]. Therefore, the increased tendency in the expression of these proteins in ruminal papillae when lambs were fed the G300 diet suggests that the use of this compound in high-concentrate finishing diets may help preserve ruminal mucosal homeostasis by preventing lesions associated with acute or subacute acidosis, thereby reducing the incidence of ruminitis.

Furthermore, the inclusion of G300 in finishing diets for lambs was associated with the expression of proteins related to reduced cellular stress and antioxidant responses. In the present study, the reduced expression of HSP70 and Annexin A1 suggests a lower cellular stress response in lambs fed crude glycerin, which may be associated with improved epithelial stability under high-concentrate feeding conditions [45]. Additionally, Rhee and Woo [43] reported that the antioxidant enzymes *Superoxide dismutase* (SOD1) and *Peroxiredoxin-6* (PRDX6) play a crucial role in neutralizing reactive oxygen species, thereby preventing oxidative damage to cellular molecules such as lipids, proteins, and DNA.

In this context, the reduced tendency in the expression of *HSP70*, *SOD1*, and *PRDX6* in ruminal papillae when lambs were fed the G300 diet may indicate the presence of an adaptive cellular protection mechanism that contributes to the maintenance of ruminal tissue health. This response is likely mediated by the combined effects of enhanced energy metabolism and proteins that reinforce epithelial barrier function.

Although economic evaluation was not included in the present study, future research should integrate performance and cost-efficiency analyses to better translate these findings into practical applications for the ruminant production industry.

## 5. Conclusions

Crude glycerin can partially replace corn in high-concentrate diets for lambs without impairing carcass yield or rumen health. Although a reduction in average daily gain was observed, the inclusion of crude glycerin promoted structural and molecular adaptations in the ruminal epithelium, including increased papillae density, enhanced epithelial activity, and modulation of proteins associated with barrier integrity, energy metabolism, and reduced oxidative stress.

These findings indicate that crude glycerin supports ruminal epithelial adaptation under high-concentrate feeding conditions and represents a viable alternative energy source in lamb finishing systems. However, the observed reduction in growth performance should be considered when defining inclusion levels in practical feeding strategies.

## Figures and Tables

**Table 1:** Ingredients and chemical composition of experimental diets.

Item	Pre-Adapt.	1st Adaptation		2nd Adaptation		3rd Adaptation		Finishing ^a^	
		CON	G300	CON	G300	CON	G300	CON	G300
Concentrated (g/kg^−1^)	240	520	520	640	640	760	760	880	880
*Ingredients* (g/kg^−1^ DM)									
Corn silage ^b^	950	450	450	350	350	250	250	150	150
Corn	-	300	-	300	-	300	-	300	-
Crude glycerin	-	-	300	-	300	-	300	-	300
Soybean hulls	15	47	10	158	120	232	193	300	281
Soybean meal	19	179	207	168	198	198	231	198	247
Urea	5	10	17	10	16	5	11	5	7
Premix mineral-vitamin ^c^	5	6	6	6	6	6	6	6	6
Limestone	4	6	4	6	4	6	4	6	4
Dicalcium phosphate	2	3	7	3	7	4	6	4	6
*Chemical composition* (g/kg^−1^ DM)									
Dry matter	334	629	638	689	698	749	758	780	817
Crud protein	94	177	177	177	177	177	177	177	177
ME, Mcal/kg ^d^	23	27	27	27	27	27	27	27	27
Ether extract	31	31	20	29	19	28	17	26	16
Neutral detergent fiber	520	346	279	381	313	393	324	395	344
Calcium	6	6	6	6	6	6	6	6	6
Phosphorus	4	4	4	4	4	4	4	4	4

^a^ Experimental diet: CON = control diet; G300 = diet with 300 g/kg of crude glycerin in dry matter. ^b^ Corn silage containing 30% grain. ^c^ Composition (g/kg): P = 75 g; Ca = 223 g; S = 10 g; Zn = 3 g; Na = 60 g; Co = 20 mg; I = 40 mg, Se = 24 mg; F = 750 mg; Mg = 5 g; Mn = 1.8 g; Fe = 402 mg; Vit. A = 312.500 IU; Vit. D = 50.000 IU; Vit. E = 437 IU. ^d^ Metabolizable energy estimated using the Small Ruminant Nutrition System (version 1.9.5105). Chemical composition values (DM, CP, NDF, and EE) were determined analytically (see Section 2).

**Table 2:** Performance of feedlot lambs fed diets containing 300 g/kg of corn (CON) or 300 g/kg of crude glycerin (G300) in the dry matter of the total mixed ration.

Item ^1^	Feedlot Period					SEM ^2^	*p*-Value ^3^		
	Day 0 (n = 5)	Adaptation (n = 20)		Finished (n = 40)					
		CON	G300	CON	G300		TRAT	TIME	TRAT × TIME
Feedlot days	5	15	15	44	54	1.92	0.12	<0.001	0.12
DMI, g/day	568	673	665	1.122	1.008	0.02	0.21	<0.001	0.12
Initial BW, kg	18.32	19.22	18.99	21.43	21.01	0.74	0.56	<0.001	0.65
Final BW, kg	18.71	21.88	21.42	35.02	34.74	0.32	0.48	<0.001	0.69
Fasting BW, kg	17.63	20.83	20.28	33.64	33.33	0.41	0.36	<0.001	0.74
ADG, kg	0.078 ^d^	0.177 ^c^	0.162 ^c^	0.321 ^a^	0.265 ^b^	0.02	0.025	<0.001	0.02
FE, g/g	137	263	244	286	262	0.03	0.12	0.045	0.45

^1^ DMI: dry matter intake; BW: body weight; ADG = average daily gain; FE = feed efficiency; ^2^ SEM = standard error of the mean; ^3^ Probability = effects: TRAT = treatment, TIME = feedlot period, and TRAT × TIME = interaction. a–d: Means within a row with different superscripts differ significantly (*p* < 0.05).

**Table 3:** Quantitative and qualitative characteristics of the carcass of feedlot lambs fed diets containing 300 g/kg of corn (CON) or 300 g/kg of crude glycerin (G300) in the dry matter of the total mixed ration.

Item	Treatments		SEM ^1^	*p*-Value ^2^
	CON (n = 20)	G300 (n = 20)		
Empty body weight, kg	33.2	33.4	0.35	0.68
Hot carcass weight, kg	17.8	17.7	0.20	0.25
Hot carcass yield, %	49.1	48.9	0.63	0.87
Cold carcass weight, kg	17.1	17.2	0.19	0.27
Cold carcass yield, %	47.6	48.2	0.56	0.36
Chilling loss, g/kg	32.1	28.3	0.28	0.07
*Longissimus* muscle area, cm^2^	14.4	14.9	0.45	0.65
Fat thickness at the 12th rib, mm	4.6 ^a^	3.7 ^b^	0.36	0.04

^1^ SEM = standard error of the mean; ^2^ Probability; ^a^, ^b^ Values within a row with different superscripts differ significantly at *p* < 0.05 (Tukey test).

**Table 4:** Stomach compartment weights, rumen lesion score, and hepatic abscess incidences in lambs fed diets containing 300 g/kg of corn (CON) or 300 g/kg of crude glycerin (G300) across different feedlot phases.

Item ^1^	Feedlot Period					SEM ^2^	*p*-Value ^3^	
	Day 0 (n = 5)	Adaptation (n = 20)		Finished (n = 40)				
		CON	G300	CON	G300		TRAT	TIME
RUM, kg	0.50	0.56	0.53	0.74	0.79	0.02	0.01	<0.001
RET, kg	0.10	0.08	0.09	0.13	0.11	0.01	0.21	<0.001
OMA, kg	0.06	0.07	0.06	0.09	0.09	0.01	0.08	<0.001
ABO, kg	0.14	0.13	0.12	0.17	0.17	0.01	0.12	<0.001
RUML, score	0.00	0.56	0.43	0.70	0.93	0.07	0.07	0.09
HA, frequency	0.00	0.58	0.00	0.00	0.00	0.04	0.04	0.11

^1^ RUM: rumen; RET: reticulum; OMA: omasum; ABO: abomasum; RUML: ruminal lesion; HA: hepatic abscess; ^2^ SEM = standard error of the mean; ^3^ Probability = effects: TRAT = treatment, TIME = feedlot period.

**Table 5:** Morphometric papillae parameters in lambs fed diets containing 300 g/kg of corn (CON) or 300 g/kg of crude glycerin (G300) across different feedlot phases.

Item ^1^	Feedlot Period					SEM ^2^	*p*-Value ^3^	
	Day 0 (n = 5)	Adaptation (n = 20)		Finished (n = 40)				
		CON	G300	CON	G300		TRAT	TIME
NOP	75.8	54.9	46.0	60.3	61.0	1.48	0.17	0.03
APA, cm^2^	0.34	0.72	0.66	0.69	0.78	0.03	0.12	0.55
ASA, cm^2^	47.2	39.5	30.1	37.8	45.2	1.75	0.33	0.09
PA, %	96.1	97.8	97.0	97.5	98.0	0.12	0.45	0.44
MIT index, %	0.49	0.90	0.95	0.98	1.10	0.02	0.11	0.01

^1^ NOP: number of papillae per cm^2^ of wall; APA: mean papilla area; ASA: total absorptive surface area; PA: area occupied by papillae relative to ASA; MIT index: mitotic index of basal cells in division; ^2^ SEM = standard error of the mean; ^3^ Probability = effects: TRAT = treatment, TIME = feedlot period.

**Table 6:** Differentially regulated proteins in ruminal papillae in lambs fed diets containing 300 g/kg of corn (CON) or 300 g/kg of crude glycerin (G300) across different feedlot phases.

Proteins ^1^	ID Uniports	Cellular Function	log_2_ FC * (G300 vs. CON)	*p*-Value ^2^	Tendency ^3^
*HSP70*	P11142	Cellular chaperone, thermal stress response	−1.2	0.02	↓
*Annexin A1*	P04083	Inflammatory response, apoptosis	−0.9	0.03	↓
*Glycerol Kinase*	Q8TAV3	Glycerol metabolism	2.3	<0.001	↑
*Claudin-1*	O14917	Epithelial barrier integrity	1.4	<0.001	↑
*Enolase-1*	P06733	Glycolysis, energy metabolism	−0.5	0.07	↓
*ATP synthase β*	P06576	Mitochondrial ATP production	0.8	0.04	↑
*Occludin*	O14976	Tight junctions, cell integrity	1	0.01	↑
*SOD1*	P00441	Antioxidant (superoxide radicals)	−1.1	0.01	↓
*Peroxiredoxin-6*	P30041	Antioxidant (peroxides and free radicals)	−0.6	0.03	↓
*Citoqueratin-4*	P19013	Epithelial structural maintenance	0.3	0.08	↔

^1^ HSP70: *Heat shock protein 70*; SOD1: *Superoxide dismutase*; ^2^ Probability; ^3^ ↓ = downregulated; ↑ = upregulated; ↔ = non-significant change expressed; * Fold Changer.

## Data Availability

The data presented in this study are available on request from the corresponding author.
